# A posterolateral sheared fracture of the tibial plateau: a case presentation

**DOI:** 10.1186/s12891-021-04373-8

**Published:** 2021-05-27

**Authors:** Jinyi Feng, Yang Gu, Wuji You, Gang Rui

**Affiliations:** 1 Department of Orthopedics Surgery, The First Affiliated Hospital of Xiamen University, 55 Zhenhai Road, Siming District, Fujian Xiamen, China; 2 Department of Trauma Orthopedics Surgery, Ningbo No.6 Hospital, Medical School of Ningbo University, 1059 Zhongshan East Road, Yinzhou District, Zhejiang Ningbo, China

**Keywords:** Posterolateral tibial plateau fracture, Long-term follow-up, Sheared fragment, Case report

## Abstract

**Background:**

Among tibial plateau fractures, one specialized type is the posterolateral column fracture. There are few published studies on posterolateral tibial plateau fractures with a sheared fragment that was wedged into the intercondylar fossa without the anterior cruciate ligament (ACL) rupture. According to our research, this case presentation is the first to describe in detail the treatment and long-term follow-up for this uncommon subtype of posterolateral tibial plateau fracture.

**Case presentation:**

A 46-year-old female injured her right knee when she was riding a motorbike and was diagnosed with a posterolateral sheared tibial plateau fracture with a wedge-shaped fragment inserted into the femoral intercondylar fossa. The fracture was repaired with open reduction internal fixation surgery. The patient’s recovery was followed for four years. The degree of healing as indicated by clinical and radiological examinations was substantial. The patient exhibited an excellent range of motion for the repaired knee (0-145°) and little discomfort. The Lysholm score was 96, the hospital for special surgery score was 98, the Rasmussen clinical assessment was 28, and the Rasmussen radiological assessment was 18.

**Conclusion:**

This study revealed that a posterolateral sheared tibial plateau, as seen in this case, can be reset and fixed sufficiently to achieve excellent long-term postoperative recovery.

## Background

Proximal tibia fracture (PTF) is the leading cause of damage to the stability and flexibility of the knee. PTF includes various subtypes and often results in loss of function. The focus for surgical repair has been to manage this fracture to achieve a joint that is stable, well-aligned, and enables an early return to normal movement and function. Several previous studies have reported that simple lateral tibial plateau fractures are relatively common and are classified as Schatzker fracture types I, II, and III, and 50 % of these fractures also include a posterolateral component [1, 2]. A posterolateral column fracture is a subtype of lateral tibial plateau fracture that commonly results when the femoral lateral condyle strikes the back of the lateral tibia plateau during flexion or partial flexion of the knee [3, 4].

Here, we report a case of a middle-aged female patient with a posterolateral tibial plateau fracture that included a sheared fragment inserted into the intercondylar fossa without the anterior cruciate ligament (ACL) rupture. This uncommon injury was treated surgically and presented excellent functional and clinical outcomes based on a long-term follow-up of four years. Based on our literature search, this case is the first to describe in detail the treatment and follow-up of this uncommon subtype of posterolateral tibial plateau fracture that cannot be easily classified into the standard classifications established by Schatzker [1].

## Case presentation

### Primary complaints

A 46-year-old female arrived at the hospital in an ambulance after hitting and injuring her right knee when riding a motorbike. The patient exhibited considerable pain in the injured knee and did not want to move it.

### History of past illness

The patient did not present any history of other notable diseases.

### Physical examination

Upon physical examination, it was observed that the patient’s right knee was red, swollen, and a skin bruise was present without any open fractures. She was unable to flex or extend her right knee due to severe pain. The patient’s body mass index was 23.7, she exhibited a Lysholm score of 0, the hospital for special surgery (HSS) score was 26, and the Rasmussen clinical assessment was 11 (Table 1).

**Table 1:** The preoperative scores and postoperative scores after four years

	Preoperative	Postoperative
*Lysholm score*		
Pain	0	25
Instability	0	25
Locking	0	15
Stair climbing	0	10
Limp	0	5
Support	0	5
Swelling	0	6
Squatting	0	5
Overall	0	96
*The hospital for special surgery (HSS) score*		
Pain	5	30
Function	0	20
ROM	4	18
Muscle strength	4	10
Flexion deformity	8	10
Instability	8	10
Subtraction	-3	0
Overall	26	98
*Rasmussen clinical assessment*		
Pain	2	6
Walking capacity	0	4
Extension	4	6
ROM	1	6
Stability	4	6
Overall	11	28
*Rasmussen radiological assessment*		
Depression	6	6
Condylar widening	2	6
Augulation (varus/valgus)	4	6
Overall	12	18

### Imaging examinations

**Fig. 1:**
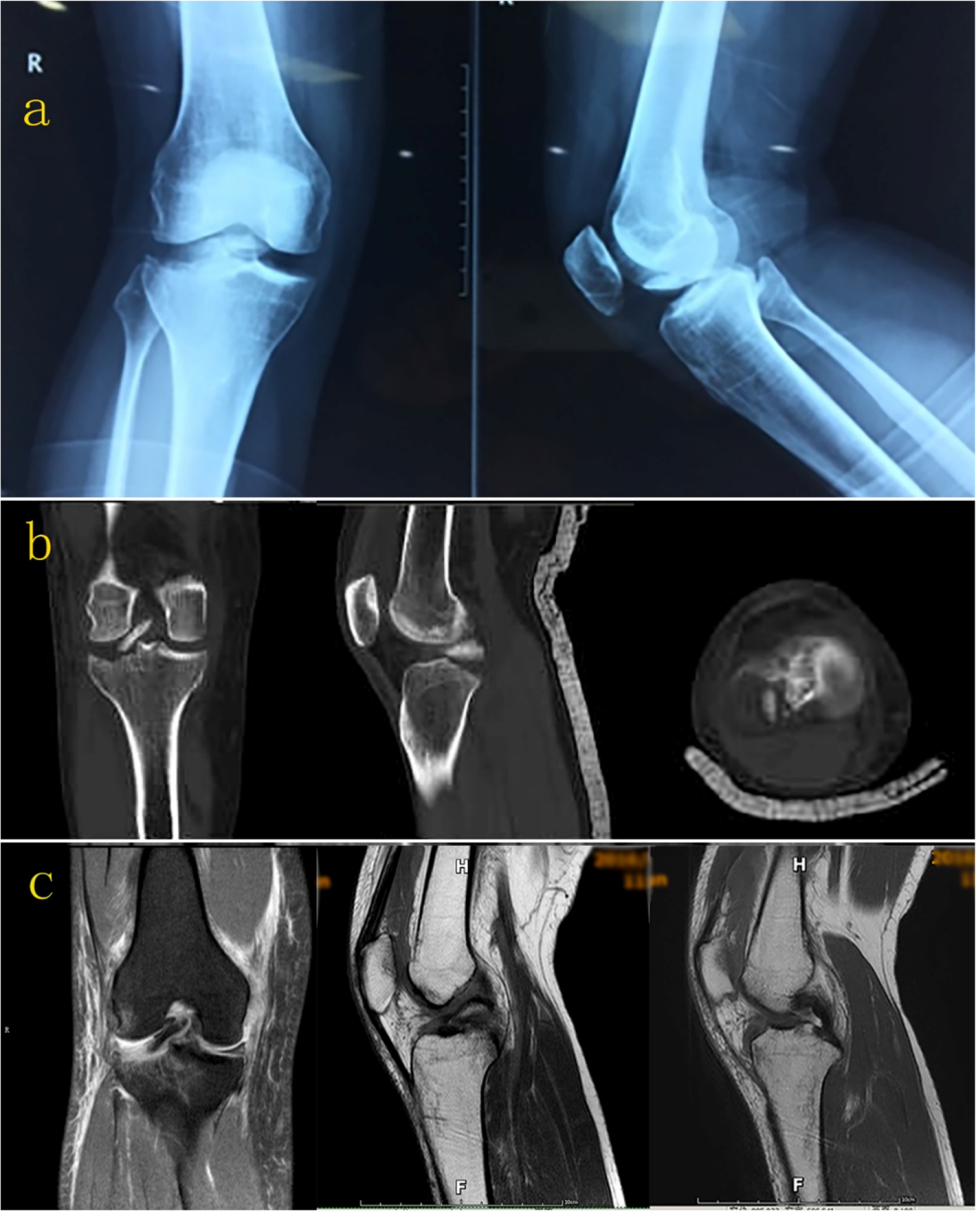
**(a)** On anteroposterior and sagittal X-rays, irregularities were observed in the lateral tibial plateau, and a high-density shadow was seen in the femoral intercondylar fossa. **(b)** CT imaging demonstrated that the lateral tibial plateau fracture was involved in a posterolateral location only, and the fragment was inserted into the femoral intercondylar fossa. **(c)** MRI showed that the ACL and PCL were intact, while the lateral meniscus and MCL exhibited injurious signal intensity

On anteroposterior (AP) X-ray, there were irregularities in the lateral tibial plateau and femoral intercondylar fossa (Fig. 1a). Imaging using computed tomography (CT) of the right knee revealed an unusual lateral tibial plateau fracture. Specifically, the damage was localized to the posterior lateral region only, and a sheared fragment was displaced into the femoral intercondylar fossa (Fig. 1b). Magnetic resonance imaging (MRI) showed that ACL and the posterior cruciate ligament (PCL) were intact with normal signal intensity, while the medial collateral ligament (MCL) and lateral meniscus presented an injurious signal intensity. (Fig. 1c). The Rasmussen radiological assessment was 12 (Table 1).

### Final diagnosis

Posterolateral sheared tibial plateau fracture.

### Treatment

**Fig. 2:**
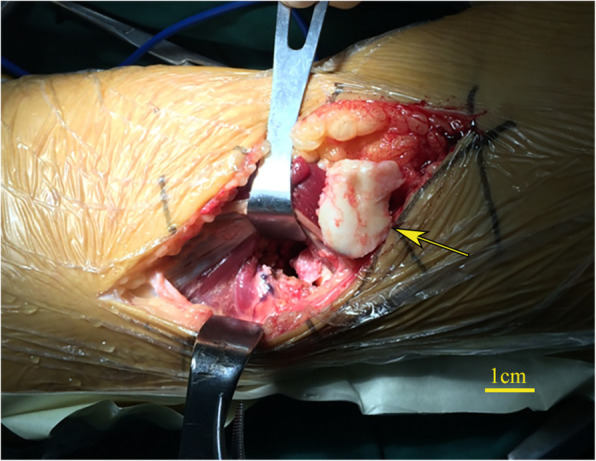
The wedge-shaped fragment was exposed during surgery

Surgery was performed using a posterior approach after the knee swelling was alleviated. In the preoperative discussion, some professors supported the assertion that the fragment was one portion of the lateral tibial plateau’s articular surface. The fragment was quite thin with limited cartilage, which led to difficulty with the repair as well as raising the possibility of avascular necrosis. To achieve full exposure and stable fixation, the patient consented to a posterior open surgical approach instead of arthroscopy. During surgery, the lateral meniscus was observed to be torn into multiple pieces beyond repair and was removed entirely. The anterior cruciate ligament and posterior cruciate ligament remained intact. The lateral condyle of the femoral articular surface was integrated and smooth. The wedge-shaped fragment was inserted in the femoral intercondylar fossa and was approximately 2 cm×2 cm×1 cm in size (Fig. 2). The thin fragment with limited subchondral bone posed difficulties in fixation. However, the stability was acceptable when the fragment was fixed with two 3.0-mm hollow screws and with the addition of 1.0-mm Kirschner wires.

On the day following surgery, the patient got out of bed and was able to use a brace designed to allow external immobilization. The patient participated in non-weight bearing and mobilized exercises for six weeks that focused on increasing her passive range of motion (ROM). The ROM at the sixth week was 10°-90°. Subsequently, the patient underwent six additional weeks of intensive physiotherapy accompanied by progressive weight-bearing.

### Outcome and follow-up

**Fig. 3:**
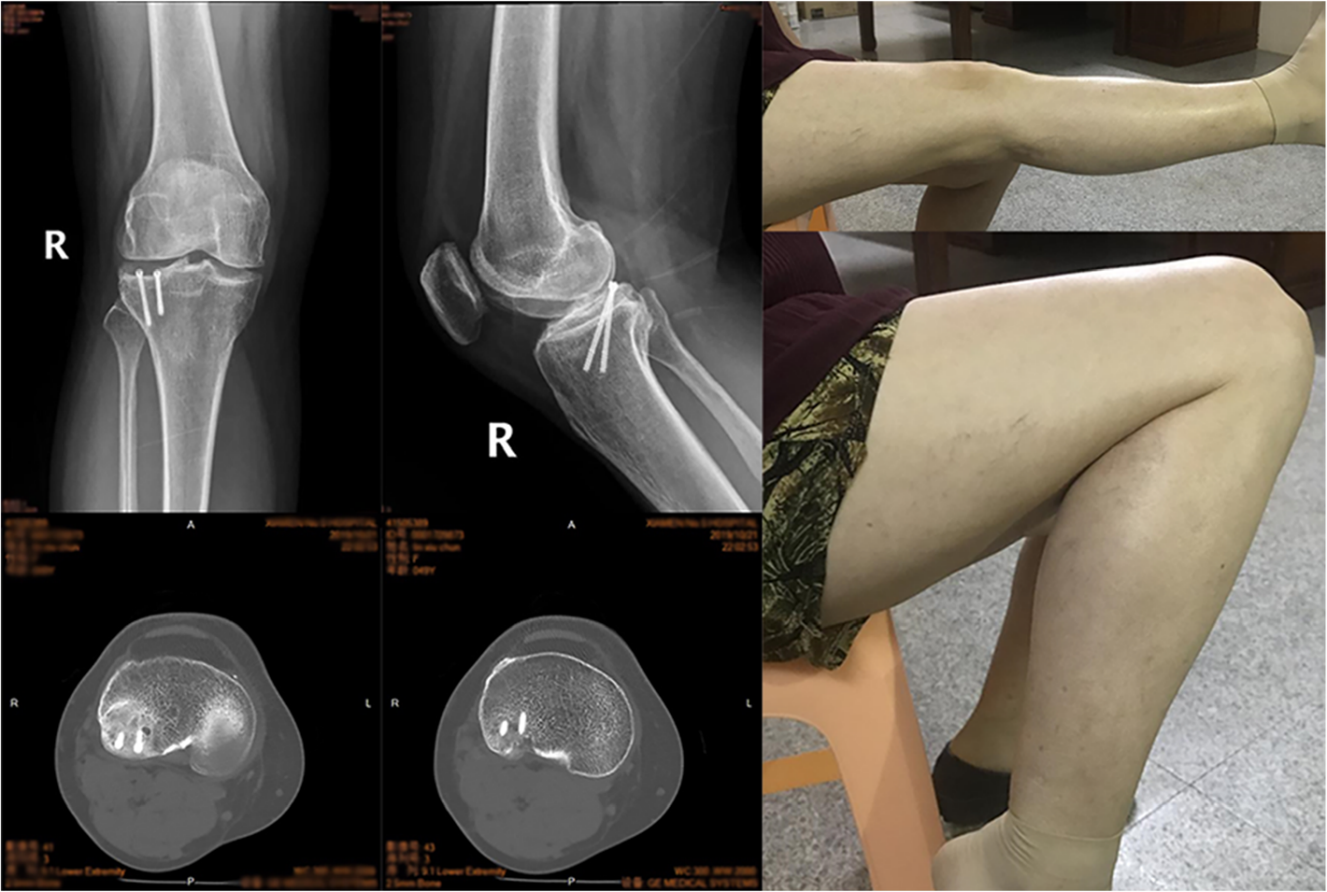
At the final fourth-year follow-up, X-ray and CT showed the fracture was healed without avascular necrosis, and a good ROM (0-145°) with limited discomfort was achieved

At the postoperative examination 12 weeks after surgery, the patient could walk without the aid of crutches. The ROM of the repaired knee had improved to 0-120°, and the radiological fracture line had disappeared. Due to the concern for avascular necrosis, follow-up examinations were prolonged to four years after surgery. At her last follow-up, the clinical and radiological evidence of healing was excellent, with a ROM of 0-145° and only limited discomfort while walking (Fig. 3). At the final follow-up, the Lysholm score was 96, the HSS score was 98, the Rasmussen clinical assessment was 28, and the Rasmussen radiological assessment was 18 (Table 1). The patient did not complain about the implant screws and did not indicate her willingness to have them removed.

#### Discussion and conclusions

This case was unusual because it presented a shear-type variation of the posterolateral tibial plateau fracture with a fragment inserted into the intercondylar fossa, which cannot be easily categorized into the standard classifications reported by Schatzker [1], Hohl [5], Moore [6], and the AO system. This case also presented a long-term follow-up of four years.

A previous case report of a male motorcyclist in the UK described a similar shear type fracture that involved the entire articular surface of the tibia and included the tibial eminence [7]. However, the tibial eminence was not involved in our case, indicating that the fragment was thinner and the fixation was more challenging. Giordano et al. [8] described these posterolateral corner sheared fractures of the tibial plateau as “apple-bite” fractures, which were rare and infrequently mentioned in previously published reports. Also, these fractures were typically associated with ACL rupture, lateral meniscus injury, and MCL tears, due to rotational trauma when the knee was in a slight valgus position and flexed [8–10]. In some cases with possible ACL rupture, it is challenging to diagnose posterolateral corner rim fractures with radiographs due to the superimposition of the larger medial condyle [11]. Thus, a CT scan and an MR imaging evaluation were highly recommended to achieve a comprehensive evaluation [8].

The tibial plateau fracture, in this case, was the result of a predominant mechanism that involved valgus stress in conjunction with a posteroanterior shear force when the knee was in the hock-flexion posture. These specific types of forces were likely to have been produced because the patient was on a motorcycle and her proximal leg struck a barrier. The MCL and lateral meniscus presented an unfavorable signal intensity as mentioned above due to rotational trauma, while the ACL and PCL were intact, which differed from conventional results. Reports by numerous studies indicate that the intra-articular aspect associated with these fractures necessitates an anatomic reduction of the fracture and good joint congruity to decrease the possible occurrence of post-traumatic osteoarthrosis [12–14]. The unusual circumstances associated with this particular injury provided striking surgical challenges. Foremost, a detailed and comprehensive understanding of fracture anatomy was necessary to determine the appropriate course of the surgical repair and plan a successful reconstruction. In this case, minimally invasive surgery with arthroscopy was considered initially but not used since the intact anterior and posterior cruciate ligaments might have blocked joint cavity enlargement to allow enough space for the posterior reset and fixation. Therefore, a minimal soft tissue dissection open surgery with a posterior approach was performed to reset the dissociative fracture bone fragment that was inserted into the femoral intercondylar fossa and fix it.

The fragment was exceedingly thin with minimal subchondral bone, which increased the risk of avascular necrosis. However, the fracture surfaces of both sides fit together well, and the fixation was stabilized with Kirchner wires. Then two hollow screws were used to stabilize the fragment. Excellent knee movement was observed and found to have minimal fixation.

Due to the possibility of early surgical intervention and stable joint congruity restoration, the fracture healed exceedingly well. No complications such as avascular necrosis were observed. During the final fourth-year follow-up, the patient exhibited an excellent surgical repair outcome. Her ROM was appropriate, and the patient did not experience any post-traumatic osteoarthrosis that affected her daily life and work.

This case described an unusual posterolateral tibial plateau fracture. Integrated radiological materials were exceedingly helpful in gaining a complete understanding of the fracture anatomy. Surgery should be considered for such injuries to obtain an adequate anatomical reduction and maintenance of the congruity of the joint. Minimizing tissue dissection as much as possible while preserving vascularity as well as anatomical and minimal fixation creates increased possibilities for a satisfactory prognosis.

In conclusion, posterolateral sheared tibial plateau fractures are rare but severe injuries. We reported on a case in which a wedge-shaped fracture fragment was reset and fixed with two screws due to the fragment’s size and specific position. The surgical repair allowed a quick return to mobility and rehabilitation for the patient. At the final fourth-year follow-up examination, the patient demonstrated excellent postsurgical recovery and exhibited considerable ROM without post-traumatic osteoarthrosis.

## Availability of data and materials

The data sets used and/or analyzed during the current study are available from the corresponding authors on reasonable request.

## Data Availability

All data generated or analyzed during this study are included in this published article.

## Abbreviations

ACL: Anterior cruciate ligament
AP: Anteroposterior
CT: Computed tomography
HSS: Hospital for special surgery
MCL: Medial collateral ligament
MRI: Magnetic resonance imaging
PCL: Posterior cruciate ligament
PTF: Proximal tibia fracture
ROM: Range of motion
